# Kiwifruit and Kiwifruit Extracts for Treatment of Constipation: A Systematic Review and Meta-Analysis

**DOI:** 10.1155/2022/7596920

**Published:** 2022-10-06

**Authors:** Mohamed Eltorki, Russell Leong, Elyanne M. Ratcliffe

**Affiliations:** ^1^Pediatric Emergency Physician, McMaster Children's Hospital, Department of Pediatrics, McMaster University, Hamilton, ON, Canada; ^2^Faculty of Health Sciences, Department of Pediatrics, McMaster University, Hamilton, ON, Canada; ^3^Pediatric Gastroenterology Physician, McMaster Children's Hospital, Department of Pediatrics, McMaster University, Hamilton, ON, Canada

## Abstract

**Introduction:**

This systematic review aimed to summarize evidence to determine the effectiveness of kiwifruit or kiwifruit extracts in the treatment of constipation.

**Methods:**

Electronic databases were searched from inception to May 2022 without any age or language limitations. Eligible studies enrolled participants with constipation who were randomized to receive kiwifruit or kiwifruit extracts vs. any nonkiwifruit control. Standardized mean difference (SMD) and mean difference (MD) with confidence intervals (CI) were determined for the following outcomes: frequency of spontaneous bowel movements (SBM), abdominal pain and straining, as well as stool type as determined by the Bristol Stool Scale (BSS). The Grading of Recommendations, Assessment, Development and Evaluations (GRADE) approach was used to rate the certainty of evidence. Our review was registered on PROSPERO (CRD42021239397).

**Results:**

Seven RCTs, including 399 participants (82% female; mean age: 42 years (SD 14.6)), were included. Compared with placebo (*n* = 95), kiwifruit extracts might increase the weekly frequency of SBM (MD: 1.36; 95% CI: −0.44, 3.16) with low certainty of evidence; moreover, it had an uncertain effect on BSS (SMD: 1.54; 95% CI: −1.33, 4.41) with very low certainty of evidence. Additionally, compared with placebo (*n* = 119), kiwifruit or its extracts reduced abdominal pain (SMD: −1.44, 95% CI −2.83, −1.66) with moderate certainty of the evidence and improved frequency of straining (SMD: −0.29; 95% CI: −1.03, 0.47). Compared with psyllium, kiwifruit may increase the weekly frequency of SBM (MD: 1.01; 95% CI: −0.02, 2.04) with moderate certainty evidence, and may increase the value on the BSS (indicating softer stools) (MD: 0.63; 95% CI: 0.01, 1.25)with low certainty of evidence. Compared to placebo, kiwifruit-encapsulated extracts may result in an increase in minor adverse events (relative risk: 4.58; 95% CI: 0.79, 26.4).

**Conclusions:**

Among individuals with constipation, there is an overall low certainty of evidence indicating that kiwifruit may increase SBM when compared to placebo or psyllium. Although overall results are promising, establishing the role of kiwifruit in constipation requires large, methodologically rigorous trials. *Protocol Registration*: PROSPERO registration number CRD42021239397.

## 1. Introduction

Chronic constipation represents a significant health burden, with a global prevalence of 14%. In North America, constipation affects 35 million Americans, with mounting economic costs related to treatments and work absenteeism. [1–5] In the UK, it affects 1 in 7 adults and 1 in 3 children, resulting in over 200,000 primary care appointments per week and an annual rise in health care costs. In 2018, the UK estimated £162 million was spent on constipation-related clinical care annually, with £87 million spent on laxatives. [6].

The two most common presentations of constipation are functional constipation (FC) and irritable bowel syndrome with constipation (IBS-C), both of which are diagnosed based on the Rome consensus clinical criteria. [7] According to Rome IV criteria, both FC and IBS-C are characterized by a decrease in the frequency of spontaneous bowel movements (SBM), a change in the appearance and consistency of stool based on the Bristol Stool Scale (BSS) [7], and straining with defecation, with abdominal pain being more predominant in IBS-C. [7].

Current common treatments for FC and IBS-C include osmotic and stimulant laxatives, fibre supplements, and probiotics. [8, 9] While most pharmacological therapies studied are superior to placebo, [8] many patients report dissatisfaction with traditional treatment options and concerns related to efficacy and safety [10]. Increasingly, patients across the age spectrum are seeking alternative, more “natural,” nonpharmacological options, with the medical community responding with increased interest in the potential benefits of food or food extracts on functional gastrointestinal disorders [11–15].

There has been increasing global recognition of kiwifruit or kiwifruit extracts being beneficial for chronic constipation management, with a growing body of literature, including the publication of several randomized controlled trials (RCTs) [16–34]. The laxative effects of kiwifruit on patients with constipation seem to be related to a combination of high fibre and water content as well as an enzyme called actinidin. Additionally, kiwifruit may improve gastrointestinal motility through the enhancement of gastric emptying and protein digestion by actinidin, as well as changes to the gut microflora and increased motility through protease-activated signaling [27]. Companies are now producing chewable capsules from freeze-dried kiwifruit that contain actinidin, plant polyphenols, dietary fibre, carbohydrates, and oligosaccharides.

While data from healthy nonconstipated volunteers has been shown to be encouraging, our research question focused on the treatment of constipation with kiwifruit or kiwifruit extracts. Specifically, our research question addressed if kiwifruit or kiwifruit extracts improve outcomes in participants who have chronic constipation, such as FC and IBS-C, including improvements in bowel movement frequency, consistency, straining, and associated abdominal pain. We aimed to summarize and critically appraise the current evidence to determine the effectiveness and safety of kiwifruit or kiwifruit extracts on the symptoms of constipation in all age groups.

## 2. Methods

### 2.1. Selection of Studies

Study eligibility criteria are outlined in Table 1. We did not employ restrictions on participant age, specific diagnosis type, or language. Our outcomes are consistent with prior published trials and systematic reviews on treatments of FC [35–38] and the core outcome sets developed and endorsed by the Rome foundation for conducting clinical trials [39, 40]. Those included (1) weekly frequency of SBM (defined as a bowel movement that is spontaneous without any rescue laxatives, suppositories, enemas, or other physical assistance and leaves a feeling of complete evacuation); (2) Rome diagnostic criteria for FC or IBS-C measured at baseline and at follow-up (time of primary outcome is assessed); (3) stool consistency according to the BSS (scale range is 1–7: 1 is hardest stools and 7 is diarrhea); (4) proportion of bowel urgency or tenesmus; (5) frequency of straining with bowel movement; (6) frequency of rescue laxatives use; (7) relief of abdominal pain; (8) adverse events reported (e.g., bloating, nausea, vomiting, and allergic reactions).

**Figure 1:**
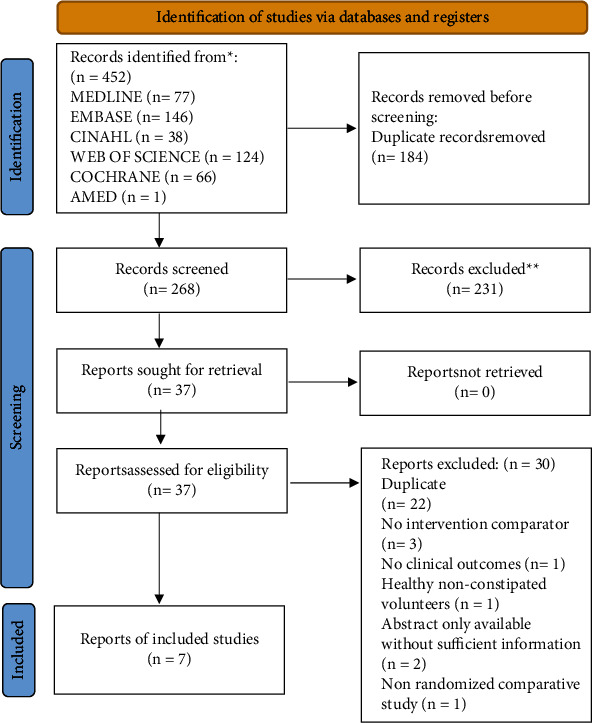
Preferred Reporting Items for Systematic Reviews and Meta-Analyses flow diagram.

Citations identified through the initial search were uploaded to a web-based systematic review management software (Covidence © 2021). [41] Using a piloted abstract screening sheet, identified abstracts were reviewed by two authors (M. E., R. L.) independently and in duplicate. Any abstract of a randomized trial that indicated the use of kiwifruit for constipation was included for full-text review. Using a piloted study selection sheet, two authors (M. E., R. L.) independently and in duplicate assessed the studies for eligibility after reading the full texts. Disagreement among authors was discussed and resolved by consensus, with a third reviewer (E. R.) acting as an arbitrator if necessary (Figure 1: PRISMA flowchart). We registered our systematic review prior to commencing the search (PROSPERO registration number CRD42021239397).

### 2.2. Search Methods for Identification of Studies

Our search strategy was developed with assistance from a medical librarian. A computer-assisted search for relevant studies (from database inception to May 18, 2022) was performed using MEDLINE, Embase, Web of Science, CINAHL, and Cochrane Library (S1 : Supplement Digital Content). References from published articles and conference proceedings were hand-searched to identify any additional citations. Abstracts were included in our search if there was a corresponding full article or if enough information was in the abstract. For abstracts without a full article, we contacted the authors for the publishing status of the full article.

### 2.3. Data Extraction and Management

A data extraction form was developed and piloted on one RCT to extract information on relevant features and results. The two reviewers independently and in duplicate abstracted data on the predefined variables on an Excel sheet and resolved any conflicts in scheduled biweekly meetings. Extracted data included the following items: author, year of publication, journal, study design, patient demographics, method of diagnosis, baseline frequency of SBM and BSS for stool type, total number of patients originally assigned to each intervention arm, interventions and comparators used including preparations, dose, administration regime, rescue therapy used, and outcomes (length of follow-up, frequency of SBM, frequency of SBM with straining, BSS for stool type, need for rescue laxatives, and number and type of adverse events associated with treatment).

### 2.4. Assessment of Risk of Bias (RoB) in Included Studies

When studies met the inclusion criteria, additional data were extracted to specifically assess the following Cochrane RoB 2.0 [42] domains: (1) adequate random sequence generation and allocation concealment; (2) masking, deviations from intended intervention, and intention to treat analysis participant; (3) missing outcome data; (4) measurement of the outcome; (5) selective reporting of the results. We used the modified Cochrane RoB 2.0 for crossover trials. A pair of reviewers (M. E., R. L.) independently assessed the articles for RoB. Response options for each item were “yes,” “probably yes,” “no,” “probably no,” and “no information.” Assignment of the final RoB assessment as “high,” “low,” or “some concerns” was done through RoB 2.0 Cochrane algorithm. If assessors' judgment differed and consensus after a discussion was not reached, a third author (E. R.) adjudicated.

### 2.5. Assessment of Certainty of Evidence

We used the GRADE (Grading of Recommendations Assessment, Development and Evaluation) approach [43] for rating the certainty of the evidence of all outcomes. We rated down the certainty based on serious (rated down by 1) or very serious (rated down by 2) concerns for the following: (1) RoB if there were overall serious or very serious concerns (not rated down if sensitivity analysis did not show an effect on overall estimates); (2) if the results were inconsistent based on inspection of the forest plot and heterogeneity; (3) if the trials included for the outcome of interest did not provide direct evidence for our research question; (4) if the results were imprecise based on total sample size <400 observations for continuous outcomes and if 95% confidence intervals (CI) excluded the null effect; (5) publication bias assessed through a funnel plot (if more than ten trials included) and by searching trial registries for unpublished trials.

### 2.6. Measures of Treatment Effect

Studies reporting on the weekly or daily frequency of SBM were pooled separately, and a mean difference (MD) with 95% CI was calculated. If studies reported on different outcome measurement scales and we were not able to impute the results on a common scale, a standardized mean difference (SMD) was calculated with a corresponding 95% CI. For dichotomous outcomes, like adverse events, we calculated a risk ratio (RR) and corresponding 95% CI. There was no established anchor-based minimally important difference (MID) for the outcomes chosen. Most clinicians and previous trials [36] used an increase of at least one weekly SBM as the MID.

### 2.7. Assessment of Heterogeneity

Heterogeneity was assessed by visual inspection of forest plots and by calculating the chi-squared test. A *p*-value for heterogeneity of <0.05 was considered significant. We also employed the *I*^2^ statistic to assess heterogeneity quantitatively. An *I*^2^ statistic of 0–40% might not be important, 30–60% represents moderate heterogeneity, 50–90% represents substantial heterogeneity, and 75–100% represents considerable heterogeneity. We assessed reasons for heterogeneity, and a sensitivity analysis was conducted if a single study was found to account for the majority.

### 2.8. Data Synthesis and Analysis

We decided a priori to analyze continuous data with a random-effects model since we expected the treatment effect would vary across studies (e.g., the effect might differ on patients with FC compared to IBS-C). We used the inverse variance to pool effect measures across continuous outcomes and Mantel–Haenszel to pool dichotomous outcomes with few events, such as adverse events. We used RevMan (Review Manager Version 5.3) [44] to perform a meta-analysis and generate forest plots. We presented our outcomes in a summary of findings table as recommended in the Cochrane Handbook for Systematic Reviews of Interventions. This was generated through GRADEPro Guideline Development Tool web-based software.

### 2.9. Subgroup and Sensitivity Analysis and Investigation of Heterogeneity

We planned to conduct the following subgroup analyses a priori, pediatric vs. adult participants and high RoB vs. low RoB. Depending on the results and the heterogeneity found between studies, we conducted an exploratory post hoc sensitivity analysis by excluding any studies with a large treatment effect and high RoB.

## 3. Results

### 3.1. Results of the Search

We identified 452 citations using the search strategy and removed 184 duplicates. After screening 268 abstracts, we retrieved 37 full-text articles to assess for eligibility (Figure 1: PRISMA flowchart). We included seven RCTs [16, 24, 25, 31, 32, 45, 46] with 399 participants. One study [46] was only reported as an abstract but had enough data on the frequency of SBM to be included. All included studies were small and individual study sample sizes ranged from 9 [16] to 120 [46] participants. All studies recruited ambulatory care patients or volunteers through advertisements. The pooled mean age (from studies reporting age) [16, 24, 25, 31, 32, 45] was 42 years (standard deviation (SD) 14.6), pooled mean weight was 62.5 kg (SD 19.2), and 82% were female (from 205/250 studies that reported gender). [16, 24, 25, 31, 45] On average across studies, baseline SBM was 2/week (SD 0.9). Four studies [16, 25, 31, 32] used encapsulated kiwifruit extracts with varying doses and three studies [24, 45, 46] used the whole kiwifruit. Four studies used placebo [16, 25, 31, 32] as a comparator and three used psyllium [24, 45, 46] as a fibre-based laxative. Given how different a placebo is from an active comparator, we performed meta-analysis and pooled results of trials that used placebo separately from trials that had an active comparator (psyllium). Duration of follow-up ranged from 2 to 16 weeks. Four studies were crossover [16, 24, 25, 46] and three were parallel-group [31, 32, 45] RCTs (Table 2: Study Characteristics).

### 3.2. Outcomes Reported

The most consistent outcomes reported were weekly or daily frequency of SBM or completed SBM, BSS, abdominal pain score, and frequency of straining. Only two studies had rescue therapy or other laxative use as an outcome, and only one provided results that showed no increase in rescue laxative use [16]. Two studies reported adverse events [25, 31]. One study used a modified BSS, [32] collapsing the 7 categories of the scale to only 3 and reversing the order of severity (1 is soft stools and 3 is very hard stool), while all other studies used the 7-point BSS.

### 3.3. RoB in Included Studies

**Figure 2:**
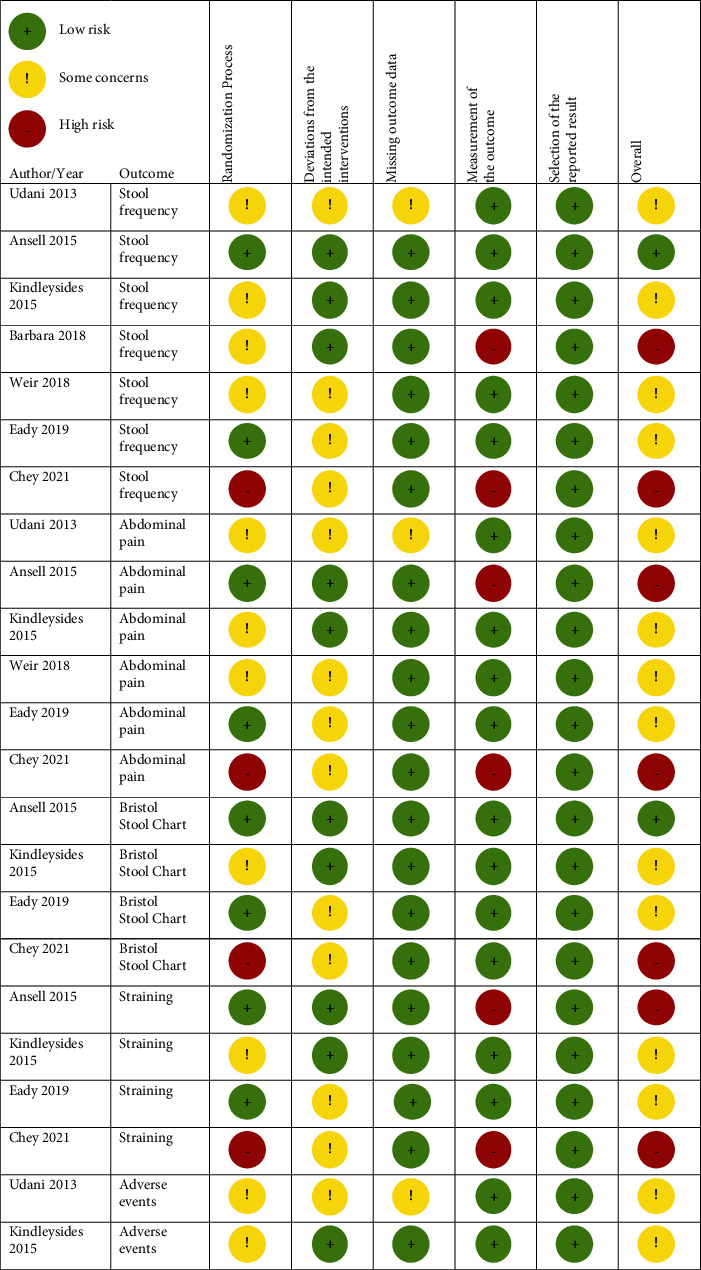
Methodological quality summary by outcome using modified Cochrane Risk of Bias 2.0 framework.

Across all outcomes, most studies had some concerns or were high RoB (Figure 2). We used the algorithm provided by Cochrane to determine the overall RoB for each study's outcome. We used overall RoB for each outcome for GRADE and presented these results in Tables 3 and 4. We were not able to construct a funnel plot to assess for publication bias since we only had seven studies.

### 3.4. Frequency of Bowel Movements

**Figure 3:**
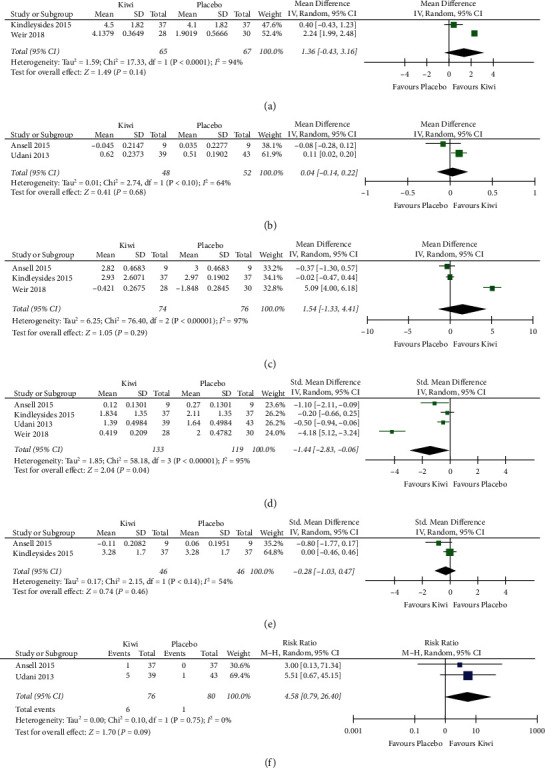
Forest plots for kiwifruits product vs. placebo. (a) Weekly frequency of spontaneous bowel movements. (b) Daily frequency of spontaneous bowel movements. (c) Bristol stool chart score (higher number indicates softer stool; Weir 2018 is negative as they reversed the scale). (d) Abdominal pain (lower score indicates improved abdominal pain). (e) Straining (lower score indicates less straining). (f) Adverse events (only available for kiwifruits vs. placebo and include bloating).

**Figure 4:**
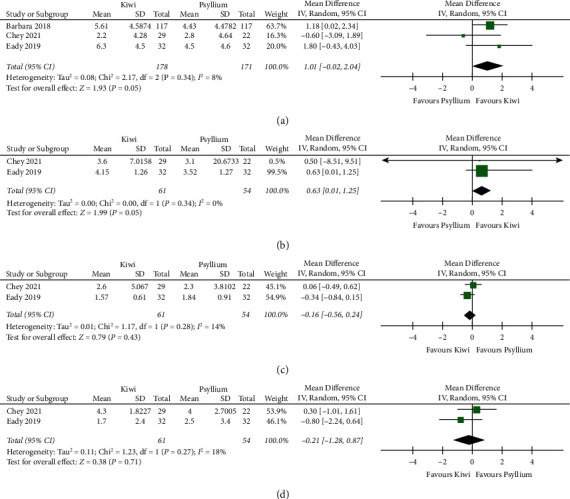
Forest plots for kiwifruits product vs. psyllium. (a) Weekly frequency of spontaneous bowel movements. (b) Bristol stool chart score. (c) Abdominal pain (lower number indicates improved abdominal pain). (d) Straining (lower score indicates less straining).

The frequency of SBM was reported in all studies included. We pooled studies reporting weekly frequency [25, 32] separately from studies that reported daily frequency of SBM [16, 31]. In two studies with 95 participants (one crossover [25] and one parallel-group RCT [32]) comparing kiwifruit to placebo, we found a mean increase of 1.36 weekly SBM favoring kiwifruit (95% CI [0.43 decrease to 3.16 increase in weekly SBM]) with low certainty of evidence (Figure 3(a), Table 3). Two studies [16, 31] (one crossover [16] and one parallel-group RCT [31]) with 91 participants comparing kiwifruit to placebo did not show a meaningful clinical difference in frequency of daily SBM, with moderate certainty of evidence (Figure 3(b), Table 3). When comparing kiwifruit (fruit or extracts) to psyllium in three studies (two crossover RCTs and one parallel-group RCT) of 200 participants [24,45], there was a mean increase of 1.01 weekly SBM (95% CI (0.02 decrease to 2.04 increase in SBM)) with moderate certainty of evidence (Figure 4(a), Table 4).

### 3.5. Bristol Stool Scale

Three of the four placebo-controlled studies, including 150 participants, reported BSS scores. When comparing kiwifruit to placebo, there was an SMD of 1.54 (95% CI: 1.33 lower to 4.41 higher) on the BSS, albeit with very low certainty of evidence (Figure 3(c), Table 3). Reasons for downgrading certainty included serious concerns with RoB, very high heterogeneity, small sample size, and a wide CI that includes the null and possible harm (worsening in BSS). In the two studies of 83 participants [24, 45] comparing kiwifruit to psyllium, participants who received kiwifruit had a higher score on the BSS (softer stool), with a mean difference of 0.63 (95% CI (0.01 higher to 1.25 higher)), with low certainty of evidence (Figure 4(b), Table 4).

### 3.6. Abdominal Pain

Four studies reported abdominal pain with 186 participants (two parallel and two crossover RCTs). Lower scores indicated clinical improvement. Kiwifruit resulted in improvement in abdominal pain with an SMD that is 1.44 lower (95% CI (2.83 lower to 0.06 lower)) than placebo (*n* = 119), with moderate certainty of evidence (Figure 3(d), Table 3). We did not downgrade for RoB since there was no subgroup effect of low vs. high-risk RoB studies on abdominal pain (Supplement 2). We downgraded by 1 for inconsistency, given a large amount of heterogeneity and the inconsistent result by Weir 2018 [32] that showed a dramatic improvement in abdominal pain compared to the rest. There was no difference in abdominal pain when comparing kiwifruit with psyllium [24, 45] (*n* = 83, one crossover and one parallel-group trial) with an SMD of 0.16 lower (95% CI (0.26 lower to 0.24 higher)), with low certainty of evidence (Figure 4(c), Table 4).

### 3.7. Straining

Two studies [24, 45] with 46 participants (two crossover RCTs) reported the weekly frequency of SBM with straining. Kiwifruit showed a small to moderate effect on straining with an SMD that was 0.28 lower than placebo (95% CI (1.03 lower to 0.47 higher)), with moderate certainty of evidence. We downgraded for imprecision given the small sample size (also explains heterogeneity) and a wide CI that includes varying effect sizes, the null, and harm (higher straining with kiwifruit) (Figure 3(e), Table 3). In the two studies comparing kiwifruit to psyllium [24, 45] with 83 participants (one parallel-group and one crossover trial), there was a small effect on straining favoring kiwifruit with 0.21 fewer episodes of weekly SBM with straining (95% CI (1.28 lower to 0.87 higher)) with low certainty of evidence (Figure 4(d), Table 4).

### 3.8. Adverse Events

Only two studies [25, 31] that used encapsulated kiwifruit extracts reported adverse events, including flatulence, bloating, nausea, or vomiting. Six participants (6/76) experienced adverse events in the kiwifruit group compared to one in the placebo group (1/80). This resulted in relative risk of 4.58 or 45 more adverse events per 1000 (95% CI (from 3 fewer to 318 more)), with low certainty of evidence.

### 3.9. Sensitivity and Subgroup Analysis

One study [32] had a substantial effect on heterogeneity of the pooled estimates comparing kiwifruit vs. placebo for SBM, BSS, and abdominal pain. Fourteen participants (19% of the sample size) dropped out of the study without any outcome data, which may have biased the results. We conducted a post hoc sensitivity analysis by excluding Weir 2018 from the pooled result, with the exception of SBM, since there were only two trials for this outcome (Supplement 3). For BSS, heterogeneity decreased from *I*^2^ 97% to 0%, and there was no change in BSS when comparing kiwifruit with placebo. For abdominal pain, heterogeneity decreased from 95% to 27%, and the SMD was reduced to 0.44 (95% CI (0.82 lower to 0.07 lower)), favoring kiwifruit. There were no studies that included children and not enough studies to conduct a subgroup for kiwifruit vs. kiwifruit extracts against the same comparator.

## 4. Discussion

Compared to placebo, kiwifruit extracts may increase the frequency of weekly SBM (moderate effect, low certainty of evidence), have little to no effect on the daily frequency of SBM (moderate certainty of evidence), have an uncertain effect on stool consistency (very low certainty of evidence), and decrease the abdominal pain and straining (moderate effect and moderate certainty of evidence). Compared to psyllium, kiwifruit may increase the frequency of weekly SBM (small important effect, moderate certainty of evidence), improve stool consistency (small important effect, low certainty of evidence), and result in little to no difference in abdominal pain or straining (trivial small effect, low certainty of evidence). Compared to placebo, kiwifruit and extracts may result in increased minor adverse events, such as bloating, flatulence, and nausea.

To our knowledge, this is the first systematic review and meta-analysis to examine the effect of kiwifruit on patients with FC or IBS-C. Although results are promising, the overall literature reflects a low certainty of evidence. As a fruit and source of fibre, there are no apparent drawbacks in adding kiwifruit to the diet, with the caveat that additional laxatives may be needed if there is no response. These findings may not be generalizable to children (not included), males (most participants were females), or severe FC (most studies only included occasional or mild FC).

The first line therapy for both FC and IBS-C in adults is bulking agents, specifically fibre; the addition of osmotic laxatives is recommended for FC but not for IBS-C [7, 9, 47]. Psyllium has been shown effective in the management of chronic constipation [48], with further meta-analyses concluding that fibre is effective in chronic idiopathic constipation [49] and IBS [9]. The potential for kiwifruit and kiwifruit extracts to be either slightly more effective or noninferior to psyllium is compelling, given the predominant role of fibre in the initial management of both FC and IBS-C. The adverse events reported with kiwifruit extracts are in line with other non-food-based laxatives and may be supportive of active laxative effects [50].

The mechanisms of action of kiwifruit are an area of the ongoing investigation. In addition to being a source of soluble and insoluble fibre, kiwifruit contain antioxidants, phytonutrients, and enzyme actinidin [27]. The actions of actinidin have been linked to digestive health, with studies suggesting that actinidin can enhance the digestion of meat proteins, dairy, and wheat (which have all been associated with IBS symptomatology) and also accelerate gastric emptying [51–53]. Given that alterations in the gut microbiota have been implicated in the pathogenesis of constipation and IBS [54–56], there has been considerable interest in the potential for kiwifruit to also act on the microbiome. For example, kiwifruit has been shown to promote the growth of beneficial microbiota for gut health, including lactobacilli and bifidobacteria [57], and also to influence the production and absorption of short-chain fatty acids [53].

Our review had several strengths and limitations. We preregistered our protocol prior to commencing data abstraction, adhered to our predefined eligibility criteria, had no language or age restrictions, and included studies reporting any outcomes. Our search was comprehensive, spanning several large databases and registries, and we used piloted screening and data abstraction forms independently and in duplicate. We assessed RoB for each outcome as per the recommendations in Cochrane Handbook for Systematic Reviews of Interventions and assessed the certainty of evidence using GRADE. There was significant heterogeneity in the trials included due to differences in design, intervention, and population, and we addressed the heterogeneity by conducting a sensitivity analysis. In addition, we were unable to perform some preplanned subgroup analyses due to a lack of sufficient information or studies, and many of our outcomes were of low certainty of evidence with low-to-moderate effect sizes.

## 5. Conclusion

Among individuals with constipation, low certainty of evidence indicates that kiwifruit may increase stool frequency and improve abdominal pain and straining when compared to placebo. Kiwifruit may improve stool frequency and improve consistency when compared to psyllium. Our findings are limited by significant heterogeneity and high RoB across trials included. Large, well-designed parallel-group RCTs comparing kiwifruit or kiwifruit extracts with known conventional and effective therapies are needed to further explore the role of kiwifruit products in the treatment of constipation.

## Figures and Tables

**Table 1:** Eligibility criteria for studies included in the systematic review.

Types of Studies	Randomized controlled trials with no language restriction
	Excluded secondary reports and conference proceedings or abstracts without sufficient information, review articles and editorials

Types of participants	Included: any age, chronic or new diagnosis of FC or IBS-C using Rome IV criteria, patient report of assessment by a physician or investigator
	Excluded: participants with constipation secondary to an underlying condition such as Hirschsprung's disease, prior bowel surgery, hypothyroidism, inflammatory bowel disease, or celiac disease

Type of interventions and comparators	Interventions: kiwifruit, kiwifruit-extracts or supplements containing kiwifruit or its enzyme actinidin, given in any form by mouth (whole fruit, capsules, chewable tablets, or powder) and at any dose for any duration of time
	Comparators: any non-kiwifruit oral laxative or placebo or no treatment

Types of outcome measures	(i) Frequency of spontaneous and/or complete bowel movements (defined as a bowel movement that is spontaneous without any rescue laxatives, suppositories, enemas or other physical assistance, and leaves a feeling of complete evacuation)
	(ii) Stool consistency according to the Bristol Stool Scale (scale range is 1-7, where 1 is hardest stools and 7 is diarrhea)
	(iii) Frequency of straining with bowel movement
	(iv) Relief of abdominal pain
	(v) Proportion of bowel urgency or tenesmus
	(vi) Frequency of rescue laxatives used
	(vii) Rome diagnostic criteria for FC or IBS-C measured at baseline and at time primary outcome is assessed
	(viii) Adverse events reported (e.g., bloating, nausea, vomiting, allergic reactions)

**Table 2:** Study characteristics.

Population					Intervention	Outcomes
Study/Design/Population	Age years (SD)	Baseline weekly frequency of spontaneous bowel movements (standard deviation)	Intervention/dose/duration	Control	Study follow-up period in days	
	Weight mean (standard deviation) kg					
	Sex (%)					
	Definition of constipation					
Weir 2018 Adults ≥18 yrs. Parallel-group double-blind RCT^*∗*^ *N* = 58	Age range: 23 to 65	2.2 (0.8)	Zyactinase capsules 2160 mg/day given as 720 mg tid	Placebo capsules containing the same color isomalt given at the same frequency as an intervention	14	(i) SBM^†^ (ii) Modified BSS^§^ (score 1–3) (iii) Abdominal pain (iv) Adverse events (nonreported)
	Not reported					
	Not reported					
	FC^#^ defined by the author					
Kindleysides 2015 Adults ≥18 yrs. Crossover double-blind RCT^*∗*^ *N* = 40	Age: 40.8 (13.2)	2.4 (0.1)	Encapsulated kiwifruit extracts containing skin, flesh, and seeds, 1000 mg/day given as 500 mg bid	Placebo was encapsulated magnesium stearate matched for weight, color, and size given at the same frequency as intervention	21	(i) SBM^†^ (ii) BSS^§^ (iii) GSRS (iv) Adverse events
	Weight 68 (14.7)					
	Female (93%)					
	FC defined by author					
Ansell 2015 Adults ≥18 yrs. Crossover double-blind RCT^*∗*^ *N* = 9	Age: 44 (6)	Not reported	Encapsulated kiwifruit extracts containing high dose Actazin 2400 mg/day given as 600 mg qid	Placebo capsules containing isomalt given at the same frequency as intervention	28	(i) SBM^†^ (ii) BSS^§^ (iii) frequency of SBM^†^ with straining (iv) incomplete evacuation (v) bloating (vi) flatulence (vii) laxatives (viii) abdominal pain
	Weight: 67 (8)					
	Female (89%)					
	Rome III FC criteria					
Udani 2013 Adults ≥18 yrs. Parallel-group double-blind placebo-controlled RCT^*∗*^ *N* = 87	Age: 39.3 (13.8)	2.1 (0.6)	Kivia powder containing zyactinase 5500 mg/day given once daily in a sachet	Placebo was powder containing inactive compounds in identical sachet to kivia given same frequency as intervention	28	(i) CSBM^◊^ (ii) BSS^§^ (iii) abdominal pain (iv) bloating (v) flatulence (vi) satisfaction with bowel habits (vii) use of rescue laxatives (viii) adverse events
	Weight: 73.5 (14.1)					
	Female (64%)					
	Rome III FC criteria					
Barbara 2018 Adults ≥18 yrs. Crossover, single-blind 3-centre RCT^*∗*^ *N* = 117	Not reported	3.5 (2.2)	Kiwifruit, 2 fruits/day	Psyllium 7.5 grams/day	14	(i) CSBM^◊^ (ii) BSS^§^ (iii) GSRS IBS-C^‡^ QoL questionnaire
Eady 2019 Adults ≥18 yrs. Crossover, single-blind, RCT^*∗*^ *N* = 35	Age: 47 (12.9)	3.0 (1.6)	Kiwifruit, 3 fruits daily	Metamucil 2.5 teaspoons (5 g of fibre total) daily	45	(i) CSBM^◊^ (ii) BSS^§^ (iii) GSRS (iv) Rome III classification
	Weight 67.5 (14.4)					
	Female (100%)					
	Rome III: (i) FC (29%) (ii) IBS-c‡ (71%)					
Chey 2021 Adults ≥18 yrs. Parallel-group nonblinded partial RCT^*∗*^ *N* = 79	Age 44.9 (16.8)	1.2 (1.5)	2 green kiwifruit daily (*Actinidia deliciosa* var. Hayward, fibre 6 g/day)	12 g of psyllium daily	28	(i) CSBM^◊^ responder rate defined as ≥1 CSBM^◊^/week (ii) CSBM^◊^ and SBM frequency (iii) BSS (iv) frequency of straining (v) abdominal pain (vi) bloating (vii) adverse events
	Weight: NR					
	Female (87%)					
	Rome III: (i) FC (71%) (ii) IBS-C (33%)					

^◊^CSBM = complete spontaneous bowel movement, GSRS = gastrointestinal symptom rating scale for abdominal pain, ^*∗*^RCT = randomized controlled trial, ^†^SBM = spontaneous bowel movement, ^#^FC = functional constipation, ^‡^IBS-C = irritable bowel syndrome with constipation, ^§^BSS = Bristol Stool Chart.

**Table 3:** Summary of findings table: kiwi compared to placebo for constipation.

Certainty assessment							No. of patients		Effect		Certainty	Importance
No of studies	Study design	Risk of bias	Inconsistency	Indirectness	Imprecision	Other considerations	Kiwi	Placebo	Relative (95% CI)	Absolute (95% CI)		
Weekly frequency spontaneous bowel movement (follow-up: range 14 to 28; assessed with: Self-reported)												
2	Randomized trials	Serious^a^	Serious^b^	Not serious	Not serious^c^		65	67	—	MD 1.36 number of bowel movements higher(0.44 lower to 3.16 higher)	⊕⊕◯◯ LOW	CRITICAL

Daily frequency of spontaneous bowel movements (follow-up: range 14 days to 28 days; assessed with: self-reported)												
2	Randomized trials	Not serious	Not serious	Not serious	Serious^d^	None	48	52	—	MD 0.04 daily SBM higher (0.14 lower to 0.22 higher)	⊕⊕⊕◯ MODERATE	CRITICAL

Stool type as per Bristol Stool Chart (follow-up: range 14 days to 28 days; assessed with: Bristol Stool Chart; scale from 1 to 7)												
3	Randomized trials	Serious^e^	Serious^f^	Not serious	Serious^g^	None	74	76	—	SMD 1.54 SD higher (1.33 lower to 4.41 higher)	⊕◯◯◯ VERY LOW	IMPORTANT

Abdominal pain (follow-up: range 14 days to 30 days; assessed with: abdominal pain scores (variable scores))												
4	Randomized trials	Not serious^h^	Serious^i^	Not serious	Not serious	None	113	119	—	SMD 1.44 SD lower (2.83 lower to 0.06 lower)	⊕⊕⊕◯ MODERATE	IMPORTANT

Straining (follow-up: range 14 days to 30 days; assessed with: different scales)												
2	Randomized trials	Not serious	Not serious	Not serious	Serious^j^	None	46	46	—	SMD 0.28 SD lower (1.03 lower to 0.47 higher)	⊕⊕⊕◯ MODERATE	IMPORTANT

Adverse events (assessed with: number of participants who experienced an adverse event)												
2	Randomized trials	Serious^k^	Not serious	Not serious	Serious^l^	None	6/76	1/80	Rate ratio 4.58 (0.79 to 26.40).		⊕⊕◯◯ LOW	NOT IMPORTANT

CI: confidence interval; MD: mean difference; SMD: standardized mean difference.(a) Studies with a high risk of bias. (b) We have clinical and methodological heterogeneity in our studies. Most of the confidence intervals do overlap, and the point estimates all favor the intervention. There is high heterogeneity in our pooled analysis, with an I^2^ of 88%. One study (Weir) is responsible for the majority of the inconsistency (I^2^ 0% if excluded). Given the high degree of unexplained heterogeneity, we have rated down for inconsistency by 1. (c) We have rated down for imprecision by 1 for the following reasons: (1) small total sample size of 232 participants, which is lower than our a priori optimal information size of 400 participants and (2) our confidence interval includes the possibility of the null and 0.5. (d) The sample size is <400 participants and the CI crosses the line of no effect. (e) RoB 2.0 for outcome stool type is serious as there are some concerns with randomization and allocation concealment in Kindleysides and Weir. (f) Weir accounts for the inconsistency in the results. Once removed, the *I*^2^ is reduced to 0%. Weir et al. collapsed the Bristol stool chart 7 types of stool to 3 categories which may have resulted in the observed large difference between kiwi and placebo in their study. (g) Total sample size for this outcome is less than 400. h. Interaction test for a subgroup for risk of bias was not significant; therefore, we did not downgrade. (i) *I*^2^ 95% and CI are not overlapping. The heterogeneity is explained by 1 study Weir 2018. They have found a dramatic improvement in abdominal pain scores that was not observed in other studies. (j) CI crosses the line of no effect (SMD 0), small sample size <400. (k) At least one domain is scored as High RoB on RoB 2.0 tool. (l) Very low number of events and wide confidence interval and RR crosses 1.

**Table 4:** Summary of findings table: kiwi compared to psyllium for constipation.

Certainty assessment							No. of patients		Effect		Certainty	Importance
No of studies	Study design	Risk of bias	Inconsistency	Indirectness	Imprecision	Other considerations	Kiwi	Psyllium	Relative (95% CI)	Absolute (95% CI)		
Weekly frequency of spontaneous bowel movements (higher number indicates an increase frequency) (follow-up: range 14 days to 30 days; assessed with: weekly mean number of SBM)												
3	Randomized trials	Serious a	Not serious b	Not serious	Not serious	None	178	171	—	MD 1.01 weekly spontaneous bowel movements higher (0.02 lower to 2 higher)	⊕⊕⊕◯ MODERATE	CRITICAL

Bristol stool scale (higher number indicates softer stool) (follow-up: range 14 days to 28 days; assessed with BSS 1–7 scale)												
2	Randomized trials	Serious a	Not serious	Not serious	Serious c	None	61	54	—	MD 0.63 BSS higher (0.01 higher to 1.3 higher)	⊕⊕◯◯ LOW	IMPORTANT

Abdominal pain (follow-up: range 14 days to 28 days; assessed with: Abdominal pain scale)												
2	Randomized trials	Serious a	Not serious	Not serious	Serious c,d	None	61	54	—	SMD 0.16 SD lower (0.6 lower to 0.2 higher)	⊕⊕◯◯ LOW	IMPORTANT

Straining frequency (lower number indicates improvement) (follow-up: range 14 days to 28 days; assessed with: weekly frequency of straining)												
2	Randomized trials	Serious a	Not serious	Not serious	Serious	None	61	54	—	MD 0.2 weekly frequency lower (1.3 lower to 0.9 higher)	⊕⊕◯◯ LOW	IMPORTANT

CI: confidence interval; MD: mean difference; SMD: standardized mean difference. (a) Chey, 2021, had major issues with randomization and allocation concealment. Included data from abstract published by Barbara, no access to the full article. (*b*) We did not rate down for inconsistency as some of the elements have been incorporated in rating down of risk of bias and imprecision. (c) Small sample size. (d) Confidence interval is wide, crosses the null, and includes harm.

## Data Availability

The data that support the findings of this study can be obtained from the corresponding author upon reasonable request.

## Supplementary Materials

Supplementary Materials Supplement 1: Search Strategy. Supplement 2: Abdominal pain (kiwi vs. placebo); with risk of bias (RoB) subgroups. Supplement 3: Sensitivity analysis. (a) Bristol Stool Chart score: higher number indicates softer stool; (b) Abdominal pain (lower number indicates improved abdominal pain). https://academic.oup.com/jcag/article/5/Supplement_1/133/6533030?login=false.
