# Supplementary material for: Kiwifruit and Kiwifruit Extracts for Treatment of Constipation: A Systematic Review and Meta-Analysis
Source: Can J Gastroenterol Hepatol. 2022 Oct 6;2022:7596920. doi: 10.1155/2022/7596920 (PMC9560827; doi:10.1155/2022/7596920)

**SUPPLEMENT 1: Search Strategy:**

CINAHL:

TX ( exp Constipation/ or exp Gastrointestinal Motility/ or Irritable Bowel Syndrome/ or Fecal Impaction/ or Defecation/ or exp Feces/ ) OR TX ( constipat* or costive* or dyschezia or colonic inertia or defecatory dysfunction* or obstipation* or rome criteria or rome I* or functional gastrointestinal disorder* or functional GI disorder* or intestinal stasis or intestinal dysmo* or intestine dysmo* or gastrointestinal dysmo* or GI dysmo* or bowel dysmo* or colon dysmo* or colonic dysmo* or rectum dysmo* or tenesmus or incomplete evacuation* or bowel evacuation* or bowel function* or gut health* or intestinal mo* or intestine mo* or gastrointestinal mo* or GI mo* or bowel mo* or colon mo* or colonic mo* or colon peristalsis or rectum mo* or irritable bowel syndrome* or IBS* or irritable colon* or spastic colon* or unstable colon* or nervous colon* or colon spasm* or colonospasm* or fecal* or faecal* or coprostasis or defecat* or defaecat* or excret* or laxat* or stool* or feces or faeces or excrement* )

TX ( Actinidia/ ) OR TX ( kiwi* or actinidia* or chinese gooseberr* or chinese egg gooseberr* or silver vine* or kivia powder* or actazin* or actinidain* or actinidin* or proteinase a2 or zyactinase* or zesy002 )

S1 AND S2

Cochrane database:

[mh Constipation] or [mh “Gastrointestinal Motility”] or [mh ^“Irritable Bowel Syndrome”] or [mh ^“Fecal Impaction”] or [mh ^Defecation] or [mh Feces]

constipat* or costive* or dyschezia or colonic inertia or defecatory dysfunction* or obstipation* or rome criteria or rome I* or functional gastrointestinal disorder* or functional GI disorder* or intestinal stasis or intestinal dysmo* or intestine dysmo* or gastrointestinal dysmo* or GI dysmo* or bowel dysmo* or colon dysmo* or colonic dysmo* or rectum dysmo* or tenesmus or incomplete evacuation* or bowel evacuation* or bowel function* or gut health* or intestinal mo* or intestine mo* or gastrointestinal mo* or GI mo* or bowel mo* or colon mo* or colonic mo* or colon peristalsis or rectum mo* or irritable bowel syndrome* or IBS* or irritable colon* or spastic colon* or unstable colon* or nervous colon* or colon spasm* or colonospasm* or fecal* or faecal* or coprostasis or defecat* or defaecat* or excret* or laxat* or stool* or feces or faeces or excrement*

or/1-2

[mh ^Actinidia]

kiwi* or actinidia* or chinese gooseberr* or chinese egg gooseberr* or silver vine* or kivia powder* or actazin* or actinidain* or actinidin* or proteinase a2 or zyactinase* or zesy002

or/4-5

3 and 6

EMBASE

exp constipation/ or abnormal feces/ or defecation disorder/ or defecation urgency/ or intestinal dysmotility/ or painful defecation/ or tenesmus/ or exp intestine motility/ or irritable colon/ or feces impaction/ or defecation/ or feces/

(constipat* or costive* or dyschezia or colonic inertia or defecatory dysfunction* or obstipation* or rome criteria or rome I* or functional gastrointestinal disorder* or functional GI disorder* or intestinal stasis or intestinal dysmo* or intestine dysmo* or gastrointestinal dysmo* or GI dysmo* or bowel dysmo* or colon dysmo* or colonic dysmo* or rectum dysmo* or tenesmus or incomplete evacuation* or bowel evacuation* or bowel function* or gut health* or intestinal mo* or intestine mo* or gastrointestinal mo* or GI mo* or bowel mo* or colon mo* or colonic mo* or colon peristalsis or rectum mo* or irritable bowel syndrome* or IBS* or irritable colon* or spastic colon* or unstable colon* or nervous colon* or colon spasm* or colonospasm* or fecal* or faecal* or coprostasis or defecat* or defaecat* or excret* or laxat* or stool* or feces or faeces or excrement*).mp.

or/1-2

exp Actinidia/ or actinidin/

(kiwi* or actinidia* or chinese gooseberr* or chinese egg gooseberr* or silver vine* or kivia powder* or actazin* or actinidain* or actinidin* or proteinase a2 or zyactinase* or zesy002).mp.

or/4-5

3 and 6

MEDLINE

exp Constipation/ or exp Gastrointestinal Motility/ or Irritable Bowel Syndrome/ or Fecal Impaction/ or Defecation/ or exp Feces/

(constipat* or costive* or dyschezia or colonic inertia or defecatory dysfunction* or obstipation* or rome criteria or rome I* or functional gastrointestinal disorder* or functional GI disorder* or intestinal stasis or intestinal dysmo* or intestine dysmo* or gastrointestinal dysmo* or GI dysmo* or bowel dysmo* or colon dysmo* or colonic dysmo* or rectum dysmo* or tenesmus or incomplete evacuation* or bowel evacuation* or bowel function* or gut health* or intestinal mo* or intestine mo* or gastrointestinal mo* or GI mo* or bowel mo* or colon mo* or colonic mo* or colon peristalsis or rectum mo* or irritable bowel syndrome* or IBS* or irritable colon* or spastic colon* or unstable colon* or nervous colon* or colon spasm* or colonospasm* or fecal* or faecal* or coprostasis or defecat* or defaecat* or excret* or laxat* or stool* or feces or faeces or excrement*).mp.

or/1-2

Actinidia/

(kiwi* or actinidia* or chinese gooseberr* or chinese egg gooseberr* or silver vine* or kivia powder* or actazin* or actinidain* or actinidin* or proteinase a2 or zyactinase* or zesy002).mp.

or/4-5

3 and 6

Web Of Science.

(constipat* or costive* or dyschezia or colonic inertia or defecatory dysfunction* or obstipation* or rome criteria or rome I or rome II or rome III or rome IV or functional gastrointestinal disorder* or functional GI disorder* or intestinal stasis or intestinal dysmo* or intestine dysmo* or gastrointestinal dysmo* or GI dysmo* or bowel dysmo* or colon dysmo* or colonic dysmo* or rectum dysmo* or tenesmus or incomplete evacuation* or bowel evacuation* or bowel function* or gut health* or intestinal mot* intestinal mob* or intestine mot* or intestine mob* or gastrointestinal mot* or gastrointestinal mob* or GI mot* or GI mob* or bowel mot* or bowel mob* or colon mot* or colon mob* or colonic mot* or colonic mob* or colon peristalsis or rectum mot* or rectum mob* or irritable bowel syndrome* or IBS* or irritable colon* or spastic colon* or unstable colon* or nervous colon* or colon spasm* or colonospasm* or fecal* or faecal* or coprostasis or defecat* or defaecat* or excret* or laxat* or stool* or feces or faeces or excrement*)

AND

(kiwi* or actinidia* or chinese gooseberr* or chinese egg gooseberr* or silver vine* or kivia powder* or actazin* or actinidain* or actinidin* or proteinase a2 or zyactinase* or zesy002)


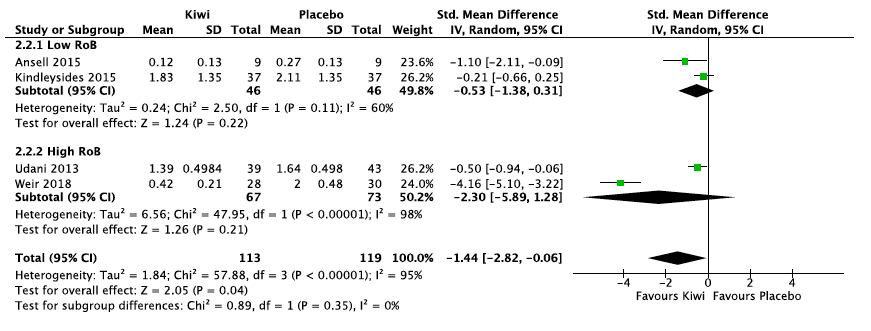
**Supplement 2: Abdominal pain (kiwi vs. placebo); with Risk of Bias (RoB) subgroups (lower number indicates improved abdominal pain)**

**Supplement 3: Sensitivity analysis**

a) Bristol Stool Chart score (higher number indicates softer stool


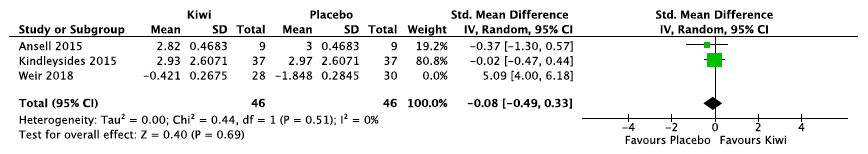


b): Abdominal pain (lower number indicates improved abdominal pain)


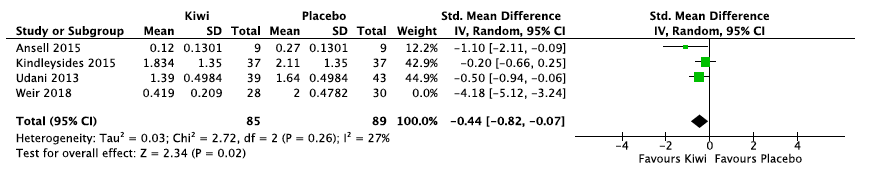

Supplement: Supplementary Materials — Supplement 1: Search Strategy. Supplement 2: Abdominal pain (kiwi vs. placebo); with risk of bias (RoB) subgroups. Supplement 3: Sensitivity analysis. (a) Bristol Stool Chart score: higher number indicates softer stool; (b) Abdominal pain (lower number indicates improved abdominal pain). https://academic.oup.com/jcag/article/5/Supplement_1/133/6533030?login=false. [file 7596920.f1.docx]
